# Supplementary material for: Empowering deep neural quantum states through efficient optimization
Source: Nat Phys. 2024 Jul 1;20(9):1476–81. doi: 10.1038/s41567-024-02566-1 (PMC11392813; doi:10.1038/s41567-024-02566-1)
Supplement: Supplementary file 1 — Supplementary Figs. 1–3 and discussion. [file 41567_2024_2566_MOESM1_ESM.pdf]

nature physics

Supplementary information https://doi.org/10.1038/s41567-024-02566-1

Empowering deep neural quantum states through efficient optimization

In the format provided by the authors and unedited

CONTENTS

A. Fubini-Study distance 2

B. Computational complexity 2

C. Structure factor 4

D. Gap estimation with and without zero-variance extrapolation 5

References 6

A. FUBINI-STUDY DISTANCE

The original definition of the Fubini-Study distance between the variational state $|\Psi_{\theta'}\rangle = |\Psi_{\theta+\delta\theta}\rangle$ and the imaginary-time evolving state $|\Psi'\rangle = e^{-\mathcal{H}\delta\tau}|\Psi_\theta\rangle$ is [1–3]

$$d(\Psi_{\theta'}, \Psi') = \arccos \frac{|\langle \Psi_{\theta'}|\Psi'\rangle|}{||\Psi_{\theta'}|| \cdot ||\Psi'||}. \tag{A.1}$$

Assuming that $|\Psi_{\theta'}\rangle = |\Psi_\theta\rangle + |\delta\Psi_\theta\rangle$ and $|\Psi'\rangle = |\Psi_\theta\rangle + |\delta\Psi_H\rangle$ where $|\delta\Psi_\theta\rangle$ and $|\delta\Psi_H\rangle$ are both small quantities, one can expand the FS distance to the lowest order of $|\delta\Psi\rangle/||\Psi||$ as

$$d^2(\Psi_{\theta'}, \Psi') = \left(\langle\delta\tilde{\Psi}_\theta| - \langle\delta\tilde{\Psi}_H|\right)\left(|\delta\tilde{\Psi}_\theta\rangle - |\delta\tilde{\Psi}_H\rangle\right), \tag{A.2}$$

where

$$|\delta\tilde{\Psi}\rangle = \frac{|\delta\Psi\rangle}{||\Psi||} - \frac{\langle\Psi|\delta\Psi\rangle|\Psi\rangle}{||\Psi||^3} \tag{A.3}$$

is the normalized increment perpendicular to the original state for $|\delta\Psi_\theta\rangle$ and $|\delta\Psi_H\rangle$.

The change of $|\Psi_\theta\rangle$ is induced by the change of parameters as

$$|\delta\Psi_\theta\rangle = \sum_\sigma \sum_k \frac{\partial\psi_\sigma}{\partial\theta_k}\delta\theta_k|\sigma\rangle = \sum_\sigma \psi_\sigma \sum_k O_{\sigma k}\delta\theta_k|\sigma\rangle, \tag{A.4}$$

where $O_{\sigma k} = \frac{1}{\psi_\sigma}\frac{\partial\psi_\sigma}{\partial\theta_k}$. According to the imaginary-time evolution $|\Psi'\rangle = e^{-\mathcal{H}\delta\tau}|\Psi_\theta\rangle$, the change of the state $|\Psi'\rangle$ to the first order of $\delta\tau$ is

$$|\delta\Psi_H\rangle = -\mathcal{H}\delta\tau|\Psi_\theta\rangle = -\delta\tau\sum_\sigma \psi_\sigma E_{\mathrm{loc},\sigma}|\sigma\rangle, \tag{A.5}$$

where $E_{\mathrm{loc},\sigma} = \sum_{\sigma'}\frac{\psi_{\sigma'}}{\psi_\sigma}H_{\sigma\sigma'}$. Putting Eq. (A.4) and Eq. (A.5) into Eq. (A.3), we obtain

$$|\delta\tilde{\Psi}_\theta\rangle = \sqrt{N_s}\sum_\sigma \frac{\psi_\sigma}{||\Psi||}\sum_k \overline{O}_{\sigma k}\delta\theta_k|\sigma\rangle, \tag{A.6}$$

$$|\delta\tilde{\Psi}_H\rangle = \sqrt{N_s}\sum_\sigma \frac{\psi_\sigma}{||\Psi||}\bar{\epsilon}_\sigma|\sigma\rangle, \tag{A.7}$$

where $\overline{O}_{\sigma k} = (O_{\sigma k} - \langle O_{\sigma k}\rangle)/\sqrt{N_s}$, $\bar{\epsilon}_\sigma = -\delta\tau(E_{\mathrm{loc},\sigma} - \langle E_{\mathrm{loc},\sigma}\rangle)/\sqrt{N_s}$.

Substituting Eq. (A.6) and Eq. (A.7) into Eq. (A.2), the FS distance becomes

$$\begin{aligned} d^2(\Psi_{\theta'}, \Psi') &= N_s\sum_\sigma \frac{|\psi_\sigma|^2}{||\Psi||^2}\left|\sum_k \overline{O}_{\sigma k}\delta\theta_k - \bar{\epsilon}_\sigma\right|^2 \\ &= N_s\left\langle\left|\sum_k \overline{O}_{\sigma k}\delta\theta_k - \bar{\epsilon}_\sigma\right|^2\right\rangle \\ &= \sum_{\sigma\,\text{in samples}}\left|\sum_k \overline{O}_{\sigma k}\delta\theta_k - \bar{\epsilon}_\sigma\right|^2, \end{aligned} \tag{A.8}$$

which proves the equation used in the main text.

B. COMPUTATIONAL COMPLEXITY

In this section, we discuss the computational complexity of NQS optimization. The epochs required for convergence can vary for different systems, so we will limit the discussion to one training iteration. The computational cost is

contributed by four major parts, namely the Monte Carlo sampling, the local energy computation, the gradient computation, and the MinSR equation. The quantities necessary for the complexity analysis are the number of lattice sites $N_{\rm site}$, the number of Monte Carlo samples N_s, the number of parameters N_p, and the order $|G|$ of the symmetry group. Furthermore, we denote the time cost of a single forward pass as F, which depends on N_p, $N_{\rm site}$, and also on the details of the chosen network architecture. The CNN architecture utilized in this work exhibits $\mathcal{O}(F) = \mathcal{O}(N_p N_{\rm site})$ [4].

The Monte Carlo sampling requires N_s symmetrized forward passes in every update, and $\mathcal{O}(N_{\rm site})$ updates to reduce the correlation between samples, leading to a complexity $\mathcal{O}(N_s\,|G|\,F\,N_{\rm site})$. For every Monte Carlo sample σ, the local energy requires computing the wave function $\psi_{\sigma'}$ for every σ' with $\langle\sigma'|\mathcal{H}|\sigma\rangle \neq 0$, which, in the typical case of local interactions, amounts to $\mathcal{O}(N_{\rm site})$ forward passes with symmetry. This again gives the complexity $\mathcal{O}(N_s\,|G|\,F\,N_{\rm site})$. To compute the gradient or the Jacobian matrix, one needs to perform a symmetrized forward and backward pass for all Monte Carlo samples. The time costs of forward and backward passes are similar, so the computational complexity is $\mathcal{O}(N_s\,|G|\,F)$. As discussed in the main text, the complexity of solving the MinSR equation is $\mathcal{O}(N_p N_s^2 + N_s^3)$. The time cost of this part is usually not a bottleneck, since the simple matrix computation is much faster than the forward pass of deep neural networks.

In summary, the dominant computational complexity, as also indicated in Extended Fig. 1(a), is $\mathcal{O}(N_s\,|G|\,F\,N_{\rm site})$ given by the Monte Carlo sampling and the local energy computation. Which one becomes the bottleneck depends on several details, including hardware memory, sample correlation, Hamiltonian connectivity, etc.

Considering $\mathcal{O}(F) = \mathcal{O}(N_p N_{\rm site})$ in the presently utilized CNN, the complexity becomes $\mathcal{O}(N_p\,N_s\,|G|\,N_{\rm site}^2)$. The dependence of N_p on $N_{\rm site}$ is, in general, an open question in the study of NQS. For the physical models considered in our work, specifically in the frustrated quantum magnets, we find that a larger N_p is required to achieve the same level of accuracy upon increasing the system size $N_{\rm site}$. Empirically, we observe that the dependence is roughly linear. However, let us point out that this likely depends on the details of the model. For our case, such an $N_{\rm site}$-dependence of N_p can be directly related to the reduction of the finite-size gap upon increasing the system size. From our own empiric observations, we find that for some models N_p can also remain unchanged, e.g., for systems with a large and finite gap. Overall, these considerations lead to an overall $\mathcal{O}(N_{\rm site}^2)$ or $\mathcal{O}(N_{\rm site}^3)$ complexity, respectively. However, let us also point out that these empirical complexity observations can also depend on the details of the chosen network architecture. In this context it is of particular importance to put a specific focus in future research to the identification of suitable architectures with improved complexity behavior in NQS.

Reducing memory complexity. The local energy and the gradient computations are bottlenecks of the memory cost. As the local energy can be computed by splitting the inputs into small batches, here we discuss how to reduce the memory complexity when computing the matrix T.

Directly computing the matrix T requires storing the value of $\overline{O}$ with shape $N_s \times N_p$, which may cause overflow issues on common GPUs for large N_p. To alleviate this problem, we compute the elements of T as

$$T_{\sigma\sigma'} = \sum_k \overline{O}_{\sigma k}\overline{O}^*_{\sigma' k} = \sum_l \sum_{k^{(l)}} \overline{O}_{\sigma k^{(l)}}\overline{O}^*_{\sigma' k^{(l)}} = \sum_l T^{(l)}_{\sigma\sigma'}, \tag{B.1}$$

where l iterates over different layers, $k^{(l)}$ iterates over parameters in layer l, and $T^{(l)}_{\sigma\sigma'} = \sum_{k^{(l)}} \overline{O}_{\sigma k^{(l)}}\overline{O}^*_{\sigma' k^{(l)}}$. Consequently, $\overline{O}$ only has to be stored for each layer instead of the whole network and its memory can be freed after obtaining $T^{(l)}_{\sigma\sigma'}$, which can be achieved by performing backward pass layer by layer. The memory complexity is hence reduced from $\mathcal{O}(N_s N_p)$ to $\mathcal{O}(N_s N_p^{(l)})$, where $N_p^{(l)}$ is the number of parameters in layer l. For more details, one can check the structured derivatives method in Ref. [5].

Another major source of memory cost comes from storing intermediate values in the computation of gradients. For a network with n_l layers and $n_y^{(l)}$ neurons in each layer, the memory complexity is $\mathcal{O}\left(n_y^{(l)} n_l\right)$ which is comparable to $\mathcal{O}(N_s N_p)$. This can be solved by the gradient checkpointing method [6], in which the intermediate values are only stored for every $\sqrt{n_l}$ layers. During the backward pass, the missing intermediate values can be recovered from the stored values. The additional cost in time only amounts to one additional forward pass, but helps to drastically reduce the memory complexity to $\mathcal{O}\left(n_y^{(l)}\sqrt{n_l}\right)$.

The aforementioned structured derivatives and gradient checkpointing methods introduce additional time costs but do not alter the time complexity. With these two methods, the memory complexity of MinSR is reduced to $\mathcal{O}\left(N_s N_p^{(l)} + n_y^{(l)}\sqrt{n_l}\right)$.

C. STRUCTURE FACTOR

There exists several literature focusing on the triangular J_1-J_2 model and supporting the existence of a quantum spin liquid (QSL) phase [7–16]. Here we also provide the spin and dimer structure factors on the 12×12 triangular lattice to confirm the existence of QSLs. The structure factor is given by

$$S(\mathbf{q}) = \frac{1}{N_{\text{site}}} \sum_{i,j} C_{ij} e^{i\mathbf{q}\cdot(\mathbf{r}_i - \mathbf{r}_j)}, \tag{C.1}$$

where C_{ij} is the spin-spin correlation $\langle \mathbf{S}_i \cdot \mathbf{S}_j \rangle$ or the dimer-dimer correlation $\langle (\mathbf{S}_i \cdot \mathbf{S}_{i+\hat{x}})(\mathbf{S}_j \cdot \mathbf{S}_{j+\hat{x}}) \rangle - \langle \mathbf{S}_i \cdot \mathbf{S}_{i+\hat{x}} \rangle \langle \mathbf{S}_j \cdot \mathbf{S}_{j+\hat{x}} \rangle$, in which we choose the dimer in x direction. As shown in Fig. 1, the spin structure peak, representing the anti-ferromagnetic (AFM) order, is weakened when J_2/J_1 increases from 0.060 to 0.125, which is expected when the system goes from an ordered phase to a QSL phase. On the other hand, the dimer structure factor representing the valence-bond solid (VBS) order remains nearly unchanged.

To further confirm the existence of a QSL phase, we measure the system-size dependence of the correlation ratio [17]

$$R = 1 - S(\mathbf{Q}_{\text{peak}} + \delta\mathbf{q})/S(\mathbf{Q}_{\text{peak}}), \tag{C.2}$$

where $\mathbf{Q}_{\text{peak}}$ represents the peak position in k-space, which is $\mathbf{Q}_{\text{peak}} = (4\pi/3, 0)$ for the spin structure and $\mathbf{Q}_{\text{peak}} = (2\pi/3, 0)$ for the dimer structure, and $\mathbf{Q}_{\text{peak}} + \delta\mathbf{q}$ is the neighbor of the peak position with a second highest structure factor. The correlation ratio of the spin structure has a crossing point at $J_2/J_1 \approx 0.063$, which can be taken as an indicator for a quantum phase transition from an AFM phase to a QSL phase. The correlation ratio of the dimer structure, instead, remains small and does not exhibit a crossing point, indicating a vanishing VBS order in the studied regime.

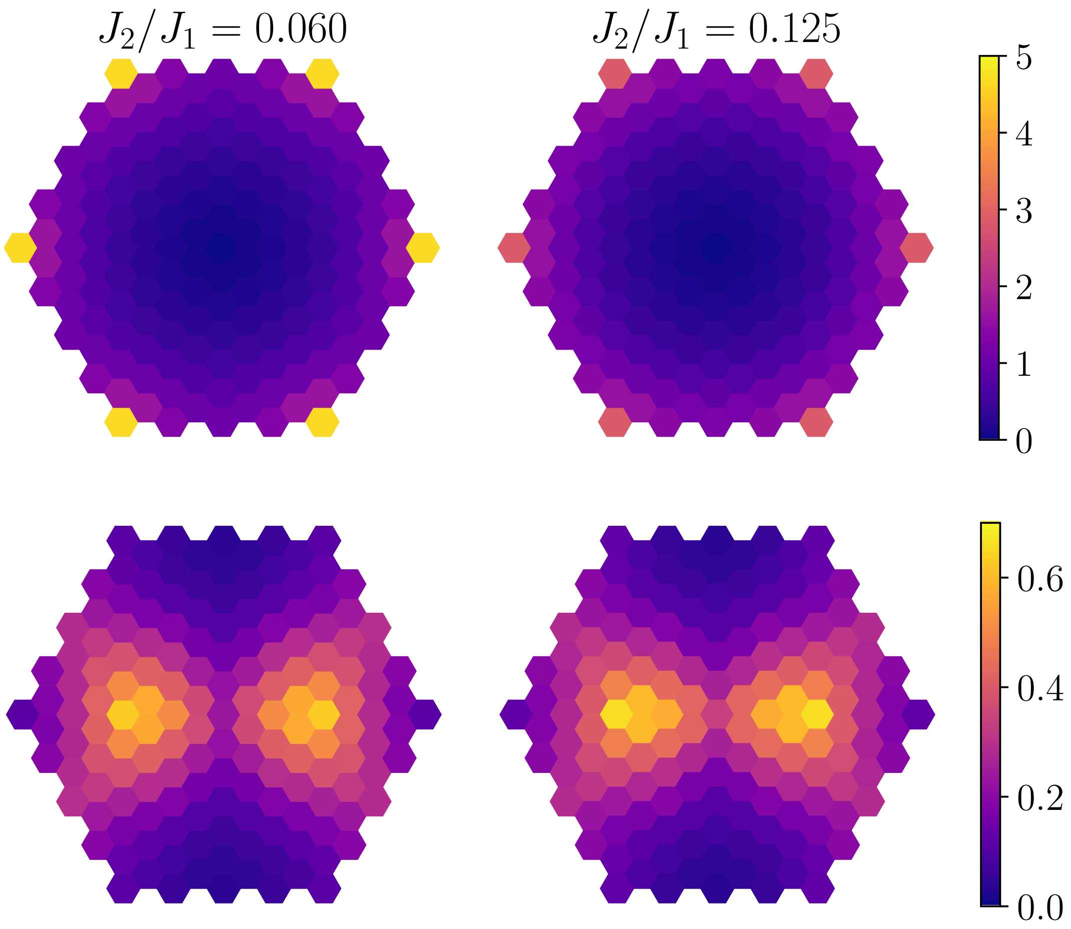

FIG. 1. The spin structure factor (upper) and the dimer structure factor (lower) in the 12×12 triangular J_1-J_2 model.

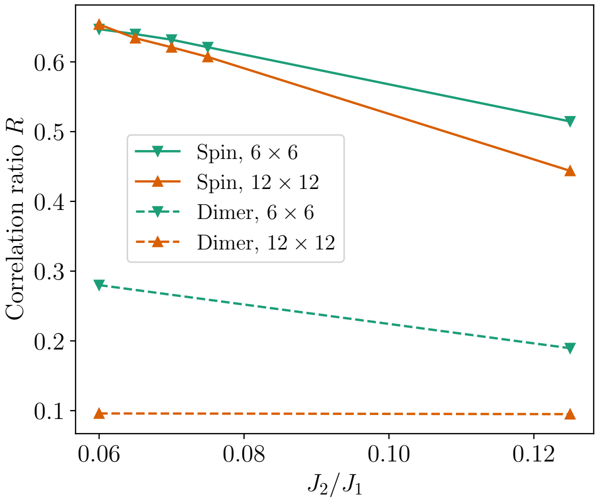

FIG. 2. The correlation ratio R of the spin and dimer structure on the 6×6 and 12×12 triangular lattice.

D. GAP ESTIMATION WITH AND WITHOUT ZERO-VARIANCE EXTRAPOLATION

In this section, we present the estimation of energy gaps with and without zero-variance extrapolation and perform a comparison between them. The result with variance extrapolation shows converged $\Delta \times L$ when $L \to \infty$, indicating $\Delta \to 0$ in the thermodynamic limit.

For the result without zero-variance extrapolation, the displayed data requires a more detailed discussion. Excluding for the moment the data point for the largest system size, our data also seems to be consistent with our previous result of a gapless phase as shown in the middle panel of Fig. 3. However, the unextrapolated 18×18 data point appears to be a complete outlier in the right panel.

The key problem in this situation is that the variational accuracy of the excited state is usually worse than that of the ground state. This difference becomes even more significant in complex systems at larger system sizes, leading to larger estimated gaps in these systems. When operating with fixed computational resources, as one increases the system size, this has the unfortunate consequence that the gap is more and more biased towards larger values. Thus, any extrapolation of such data to the thermodynamic limit suffers in this sense from a systematic bias toward larger

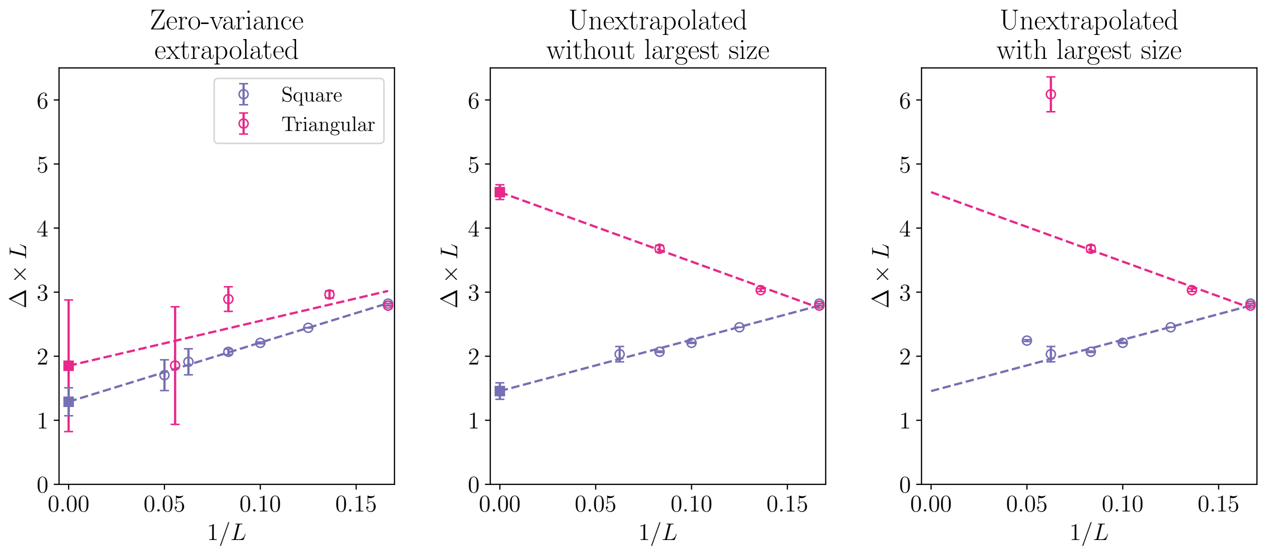

FIG. 3. The system size dependence of the energy gaps in the triangular J_1-J_2 model at $J_2/J_1 = 0.125$.

values.

Although this deviation may not apply to all different circumstances, it is still quite common in the variational Monte Carlo. For instance, in Ref. [18] the overestimation of energy gaps is observed when the zero-variance extrapolation is not performed. In particular, when aiming at deciding whether a gap closes or not, it is of key importance to eliminate this bias, which requires an extrapolation as we have been performing.

[1] G. Fubini, *Sulle metriche definite da una forma hermitiana: nota* (Office graf. C. Ferrari, 1904).
[2] E. Study, Kürzeste wege im komplexen gebiet, Mathematische Annalen **60**, 321 (1905).
[3] C.-Y. Park and M. J. Kastoryano, Geometry of learning neural quantum states, Phys. Rev. Research **2**, 023232 (2020).
[4] K. He and J. Sun, Convolutional neural networks at constrained time cost, in *Proceedings of the IEEE Conference on Computer Vision and Pattern Recognition (CVPR)* (2015).
[5] R. Novak, J. Sohl-Dickstein, and S. S. Schoenholz, Fast finite width neural tangent kernel, in *International Conference on Machine Learning* (2022).
[6] T. Chen, B. Xu, C. Zhang, and C. Guestrin, Training deep nets with sublinear memory cost (2016), arXiv:1604.06174 [cs.LG].
[7] R. Kaneko, S. Morita, and M. Imada, Gapless spin-liquid phase in an extended spin 1/2 triangular heisenberg model, Journal of the Physical Society of Japan **83**, 093707 (2014).
[8] Z. Zhu and S. R. White, Spin liquid phase of the $s = \frac{1}{2}$ $J_1 - J_2$ heisenberg model on the triangular lattice, Phys. Rev. B **92**, 041105 (2015).
[9] W.-J. Hu, S.-S. Gong, W. Zhu, and D. N. Sheng, Competing spin-liquid states in the spin-$\frac{1}{2}$ heisenberg model on the triangular lattice, Phys. Rev. B **92**, 140403 (2015).
[10] Y. Iqbal, W.-J. Hu, R. Thomale, D. Poilblanc, and F. Becca, Spin liquid nature in the heisenberg $J_1 - J_2$ triangular antiferromagnet, Phys. Rev. B **93**, 144411 (2016).
[11] S. N. Saadatmand and I. P. McCulloch, Symmetry fractionalization in the topological phase of the spin-$\frac{1}{2}$ $J_1 - J_2$ triangular heisenberg model, Phys. Rev. B **94**, 121111 (2016).
[12] A. Wietek and A. M. Läuchli, Chiral spin liquid and quantum criticality in extended $s = \frac{1}{2}$ heisenberg models on the triangular lattice, Phys. Rev. B **95**, 035141 (2017).
[13] S.-S. Gong, W. Zhu, J.-X. Zhu, D. N. Sheng, and K. Yang, Global phase diagram and quantum spin liquids in a spin-$\frac{1}{2}$ triangular antiferromagnet, Phys. Rev. B **96**, 075116 (2017).
[14] S. Hu, W. Zhu, S. Eggert, and Y.-C. He, Dirac spin liquid on the spin-1/2 triangular heisenberg antiferromagnet, Phys. Rev. Lett. **123**, 207203 (2019).
[15] Y.-F. Jiang and H.-C. Jiang, Nature of quantum spin liquids of the $s = \frac{1}{2}$ heisenberg antiferromagnet on the triangular lattice: A parallel dmrg study, Phys. Rev. B **107**, L140411 (2023).
[16] N. E. Sherman, M. Dupont, and J. E. Moore, Spectral function of the $J_1 - J_2$ heisenberg model on the triangular lattice, Phys. Rev. B **107**, 165146 (2023).
[17] Y. Nomura and M. Imada, Dirac-type nodal spin liquid revealed by refined quantum many-body solver using neural-network wave function, correlation ratio, and level spectroscopy, Phys. Rev. X **11**, 031034 (2021).
[18] W.-J. Hu, F. Becca, A. Parola, and S. Sorella, Direct evidence for a gapless Z_2 spin liquid by frustrating néel antiferromagnetism, Phys. Rev. B **88**, 060402 (2013).
